# Bilateral adrenal hemorrhage due to heparin-induced thrombocytopenia following partial nephrectomy – a case report

**DOI:** 10.12688/f1000research.3-24.v1

**Published:** 2014-01-23

**Authors:** Ashley G. Winter, Ranjith Ramasamy

**Affiliations:** 1 Department of Urology, New York Presbyterian Hospital, Weill Medical College of Cornell University, New York, 10065, USA; 2 Department of Urology, Baylor College of Medicine, Houston, Texas, 77030, USA

## Abstract

Heparin-induced thrombocytopenia (HIT) can cause severe life-threatening events such as bilateral adrenal hemorrhage (BAH). A 48-year-old female developed a pulmonary embolus (PE) following partial nephrectomy. The anticoagulation treatment for her PE was complicated by HIT and subsequent BAH. To the author’s knowledge, this is the first reported case of HIT-associated BAH following renal surgery.

## Case presentation

A 48-year-old Caucasian female with a past medical history of hypothyroidism presented to us with an incidentally discovered 3 cm enhancing right renal mass. Her home medications included levothyroxine and oral contraceptives. She underwent an uncomplicated robotic-assisted laparoscopic right partial nephrectomy. The patient received routine perioperative deep venous thrombosis (DVT) prophylaxis with sequential compression devices, (SCD). On postoperative day (POD) 2, she developed new-onset sinus tachycardia and showed depressed pulse-oximetry readings. As subsequent chest computed tomography (CT) scans demonstrated multiple large pulmonary emboli, she was started immediately on a continuous intravenous unfractionated heparin infusion, titrated to target an activated partial thromboplastin time (aPTT) of 64–97 seconds. The patient was transitioned to therapeutic anticoagulation with low-molecular-weight heparin (LMWH) at 1mg/Kg q12hrs on POD 3 and discharged the next day.

On postoperative day 6, she developed syncope and hypotension and was admitted to the hospital. Work up in the emergency room was remarkable for a hematocrit (HCT) of 19%, and a CT scan demonstrated a bleeding pseudoaneurysm in the nephrectomy bed. She responded to red blood cell transfusion (4 units) and embolization of the pseudoaneurysm, becoming normotensive with a HCT of 26.2%. Anticoagulation with LMWH at 1mg/Kg was resumed the next day. She was discharged from the hospital on POD 10.

**Figure 1.**
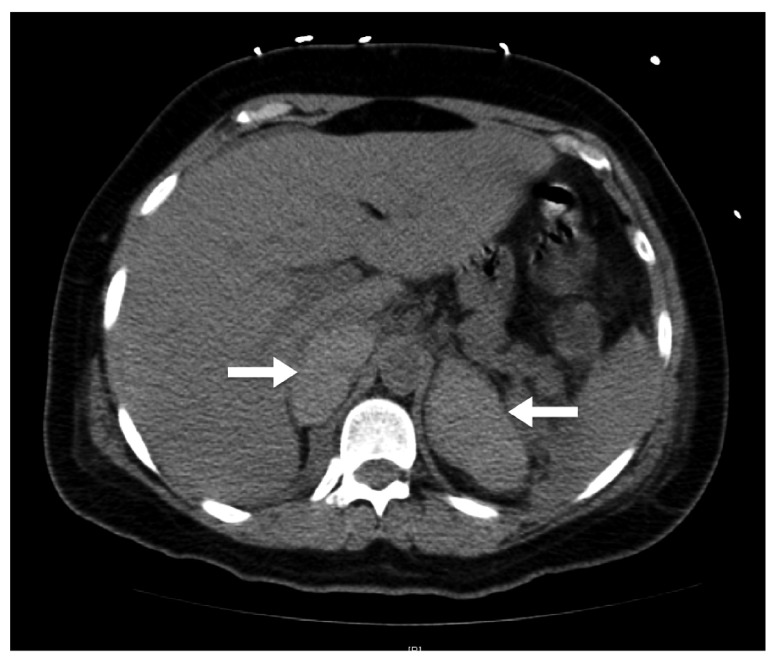
Non-contrast computed tomography scan of adrenal glands. CT scan of adrenal glands; white arrows indicate severely enlarged, hyperdense, adrenal glands bilaterally consistent with adrenal hemorrhage.

The patient presented to the surgeon’s office on postoperative day 13 with progressive lethargy and subjective fevers. She was normotensive but tachycardic, and was admitted with concern for recurrent hemorrhage. Laboratory analysis at that time was remarkable for a leukocytosis to (16,000/µL), thrombocytopenia of 49,000/µL (from a pre-operative value of 234,000/µL), hyponatremia to 125mEq/L (baseline of 139mEq/L) and a relatively normal HCT of 31.7%. CT scan of the adrenal glands demonstrated bilateral adrenal gland enlargement and hyperdensity, consistent with BAH ( Figure 1). Within 2 hours of initiating 50 mg intravenous hydrocortisone every 8 hours, her pulse decreased from 128 beats per minute (bpm) to 107bpm, and her blood pressure increase from 89/54mmHg 115/63mmHg. A percutaneous supra-renal inferior-vena-cava filter (IVCF) was placed by interventional radiology and an argatroban infusion started. The patient underwent careful titration to an outpatient regimen of fludrocortisone, hydrocortisone, and fondaparinux. She was discharged with a platelet count of 141,000/µL and serum sodium of 139mEq/L. After 12 months follow-up, the patient remains on non-heparin anticoagulation and steroid supplementation.

## Discussion

Heparin-induced thrombocytopenia is an immune-mediated complication occurring infrequently following exposure to unfractionated or low-molecular weight heparin. HIT occurs when a complex between IgG antibodies and heparin-platelet-factor-4 forms. The antibodies activate platelets by binding to the FcɣIIa receptor, which results in systemic thrombosis and consumptive thrombocytopenia ^1^.

With their rich arterial supply and single central vein, the adrenal glands are particularly susceptible to congestive hemorrhage following venous thrombosis. Adrenal hemorrhage associated with HIT tends to be bilateral, resulting in severe clinical manifestations from abdominal pain, fever and lethargy, to catastrophic hemodynamic collapse ^2^. In the post-surgical patient, these symptoms can be confused with more common complications, such as sepsis or hemorrhage. As undiagnosed cases tend to be fatal, a prompt recognition is crucial ^2^.

Few cases of HIT-associated BAH have been previously described in the literature ^2– 4^. Approximately 90% of the patients are post-surgical, with the most common operations being orthopedic. HIT may be an important cause of BAH, accounting for up to 10% of the cases ^4^. To our knowledge, this is the first reported case of HIT-associated BAH following partial nephrectomy in particular, or urologic surgery in general. Our case highlights two critical points; 1) the diagnostic complexity of HIT-associated BAH, and 2) the possibility of a successful clinical outcome when rapid diagnosis is made.

## Consent

Written informed consent for publication of clinical details was obtained from the patient.
