# Supplementary material for: Bilateral adrenal hemorrhage due to heparin-induced thrombocytopenia following partial nephrectomy – a case report
Source: F1000Res. 2014 Jan 23;3:24. [Version 1] doi: 10.12688/f1000research.3-24.v1 (PMC3954162; doi:10.12688/f1000research.3-24.v1)
Supplement: Peer review history

# Peer review: Referee response for version 1

**Published:** 2014-02-24

**Authors:** Boback Berookhim

**Affiliations:** 1 Memorial Sloan Kettering Cancer Center, New York, NY, USA

The authors provide a report of an interesting clinical situation following robotic assisted partial nephrectomy. The difficulties of diagnosis and non-specific presentation of potentially life-threatening bilateral adrenal hemorrhage in a patient with previously diagnosed pseudoaneurysm are clearly spelled out. An excellent literature review is provided. This article serves as a reminder to the urologist of the untoward and life threatening potential effects of heparin induced thrombocytopenia, and the importance of prompt diagnosis and treatment of BAH.

I have read this submission. I believe that I have an appropriate level of expertise to confirm that it is of an acceptable scientific standard.

# Peer review: Referee response for version 1

**Published:** 2014-01-24

**Authors:** Ryan Smith

**Affiliations:** 1 Department of Urology, University of Virginia Medical Center, Charlottesville, VA, USA

The authors have composed an interesting case report with a good review of the literature. Anticoagulation requirements before and after Urologic surgery have significant importance and can result in a myriad of post-operative complications. Prompt recognition of these is essential to prevent additional patient morbidity. HIT-associated BAH represents a less commonly seen complication following anticoagulation and it is important for surgeons to be aware of this possibility.

I have read this submission. I believe that I have an appropriate level of expertise to confirm that it is of an acceptable scientific standard.
